# Visualization is crucial for understanding microbial processes in the ocean

**DOI:** 10.1098/rstb.2019.0083

**Published:** 2019-10-07

**Authors:** Marta Sebastián, Josep M. Gasol

**Affiliations:** 1 Instituto de Oceanografía y Cambio Global, IOCAG, Universidad de Las Palmas de Gran Canaria (ULPGC), Spain; 2 Institut de Ciències del Mar, CSIC, Barcelona, Catalunya, Spain; 3 Centre for Marine Ecosystems Research, Edith Cowan University, Joondalup, Western Australia, Australia

**Keywords:** single-cell activity, growth, microbes, visualization, ocean

## Abstract

Recent developments in community and single-cell genomic approaches have provided an unprecedented amount of information on the ecology of microbes in the aquatic environment. However, linkages between each specific microbe's identity and their *in situ* level of activity (be it growth, division or just metabolic activity) are much more scarce. The ultimate goal of marine microbial ecology is to understand how the environment determines the types of different microbes in nature, their function, morphology and cell-to-cell interactions and to do so we should gather three levels of information, the genomic (including identity), the functional (activity or growth), and the morphological, and for as many individual cells as possible. We present a brief overview of methodologies applied to address single-cell activity in marine prokaryotes, together with a discussion of the difficulties in identifying and categorizing activity and growth. We then provide and discuss some examples showing how visualization has been pivotal for challenging established paradigms and for understanding the role of microbes in the environment, unveiling processes and interactions that otherwise would have been overlooked. We conclude by stating that more effort should be directed towards integrating visualization in future approaches if we want to gain a comprehensive insight into how microbes contribute to the functioning of ecosystems.

This article is part of a discussion meeting issue ‘Single cell ecology’.

## 1. Introduction

Understanding the ecology of environmental microbes requires collecting information on the diversity or identity of microbes, their role in the natural environment, how the different microbes interact among themselves and with larger organisms, and how all this is affected by environmental variability. The advent of molecular techniques has revolutionized the field of environmental ecology, providing a tremendous boost to our understanding of microbial processes, but the ultimate goal should be to develop methods that can tell us the identity of microbes with their corresponding level of activity, and that provide information about their size, complexity and behaviour on an individual cell-basis. Because we are interested in ecology, and microbial communities are complex, we would need to obtain this information from as many cells as possible, something that facilitates proper statistical testing of ecological hypotheses.

In any ecosystem, bacterial cells occur in a continuum of metabolic states: dead or injured, dormant or non-growing, metabolically active but limited by one or more essential nutrients, or actively growing. Active cells are those driving ecosystem processes, and thus maintain the ecosystem function potential [1], growing cells increase the ecosystem function, and inactive cells can be considered a repository of ecosystem functions [2], as they contain all the functional capabilities of the community and, therefore, can provide ‘ecological insurance’ to the ecosystem facing change [3]. On the other hand, dead cells imply a loss of ecosystem function, but they can persist for a long time in the environment and become a source of energy and nutrients, and also be a source of genetic innovation through natural transformation [4]. As a result, the metabolic state of bacterial cells in the environment has profound ecological implications, and scientists have pondered over this issue since the importance of bacteria in the marine food web was recognized (e.g. [5]). However, the level of activity of an individual cell, referred to as its ‘single-cell activity’, is understood in multiple ways by different researchers depending on the targeted functional trait and the methodology employed, as depicted below.

**Figure 1.**
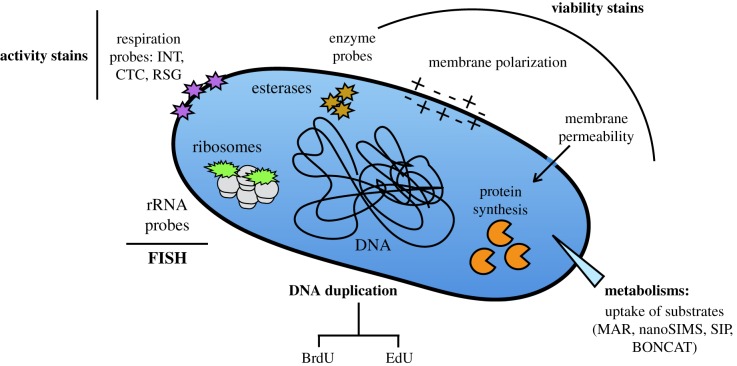
Schematic of a prokaryotic cell with indication of some of the methods used to probe cellular activity or growth. *Viability probes* include a large variety of stains (e.g. propidium iodine, PI; or DiOC(3)) that can be used to investigate the state of the bacterial membrane, membrane electrical polarization or potential (e.g. [6]). Another set of probes target the intracellular enzymes, most notably the activity of intracellular esterases (e.g. Calcein Blue) or intracellular pH (e.g. the SNARF series of stains). The relative properties of the nucleic acids can be detected using stains such as acridine orange, Syto or SybrGreen; or by de-staining after 4′,6-diamidino-2-phenylindole (DAPI) [7]. Closer to cell metabolism, there is an assortment of activity stains, like those targeting the enzymatic processes in the electron transport respiratory chain, as for example 2-(4-iodophenyl)-3-(4-nitrophenyl)-5-phenyl tetrazolium chloride (INT), 5-cyano-2,3-ditolyl tetrazolium chloride (CTC), [8,9]) or Redox Sensor Green (RSG) [10]. Other approaches involve the detection of the uptake of certain substrates. For example, microautoradiography (MAR) allows detection of cells active in the uptake of a variety of radioactive substrates, and is possible to quantify the uptake rates for each specific cell (e.g. [11]). Similarly, nanoscale secondary ion mass spectrometry (nanoSIMS) and RAMAN-microscopy allow detection and quantification of the incorporation of stable-isotope labelled substrates [12,13]. In the past, the fluorescent signal of fluorescence *in situ* hybridization (FISH) was considered an estimator of physiological status [14], but with the amplification of the signal with catalysed reporter deposition (CARD-FISH) the use of this technique is currently limited to the identification of different phylogenetic groups. DNA duplication can be measured using thymidine analogues that detected immunochemically (BrdU [15]) or by other means (EdU [16]), and protein synthesis can also be detected using specific synthetic amino acids (bioorthogonal non-canonical amino-acid tagging, BONCAT [17]). Figure updated from del Giorgio & Gasol [18].

One of the simplest approaches to assess the physiological status of a bacterial cell is to account for whether the cell is active or inactive in a given biogeochemical process: e.g. whether the cell is respiring, or whether the cell is photosynthesizing, or taking up a specific compound (figure 1; see also [18,19] for a detailed overview on this topic). Another approach is to consider whether the cell is actively growing, but here things might get more complex because growth can be defined by division or by biomass production, which does not necessarily indicate division. In addition, cells can be metabolizing (i.e. respiring) yet not actively growing. Another way is to probe whether the cell is intact or shows signs of degradation, e.g. has lost membrane potential, or has a leaky membrane (figure 1). While some of the methods used to assess the metabolic state are qualitative, others are more or less quantitative, i.e. with the needed calibrations a certain level of metabolism can be assigned to each of the cells, and some methods can be combined with other tools to gather phylogenetic information (as we will expand below).

Given that natural planktonic bacteria sometimes have levels of activity that are below or in the range of the methods' detection, and that the different methods target different processes, none of the ‘active’ ‘inactive’ ‘dead’ or ‘growing’ categories can in practice be well defined, and they are basically operational depending on the method used (e.g. [20]). That the methods might not offer absolute results does not impede their use to answer ecologically relevant questions, in which a comparative approach is still valid. As an example, microautoradiography (MAR) combined with fluorescent *in situ* hybridization (FISH) has been used to describe which bacterial groups are more limited by an inorganic nutrient [21], or to quantify substrate uptake by individual cells [11], or the relative contribution of different phylogenetic groups to the use of dissolved organic matter or inorganic nutrients [22,23].

Yet, there are several issues regarding the use of most single-cell methods that are worth mentioning because they illustrate some of the problems that researchers might encounter in their daily use and interpretation. One obvious one, but not always taken into account, is that the affinity for the substrate plays a role in the level of activity we detect (e.g. [24]). For example, marine Flavobacteria display very low levels of activity compared with other groups when amino acids are used as substrate in MAR assays, because they have a preference for high molecular weight compounds [22]. So if using amino acids to evaluate the metabolic state of Flavobacteria and quantifying their contribution to carbon flow, we would obtain biased results. Similarly, method interpretation might also be biased by the observation device. For example, 5-cyano-2,3-ditolyl tetrazolium chloride (CTC) is an activity stain that has been used to detect and quantify the cells that are actively respiring in a bacterial community (figure 1). The method was first used with microscopy, but soon flow cytometry was incorporated as an efficient approach to quantify the distribution of respiration activities within a population with statistical confidence [25]. A few years later, the method raised criticism because CTC was seen to be toxic in natural communities, inhibiting protein synthesis [26,27]. Indirect visualization through flow cytometry was key to unveiling that the formazan salt granules produced upon reduction of CTC ended up breaking the cells [28] but the granules were still indicating the respiratory activity, thus resolving the contradiction between the toxic effect observed and the method reliability to estimate the proportion of actively respiring cells if incubation times were kept low. These examples reiterate the need to experimentally determine the accuracy of the cell-specific activity method of choice for microbes in their natural environment.

Many of the single-cell methods shown in figure 1 seem to have lost popularity in the last years, perhaps owing to both the lack of certainty about what process they are measuring and also as a consequence of the blooming of high-throughput sequencing technologies. However, the era of descriptive science based on ‘omic and diversity data’ is probably over, and right now we should be moving towards a more hypothesis-driven science, which may result in the comeback of some of these single-cell methods, particularly those that can be coupled with downstream analyses for molecular characterization, as detailed in the next section.

## 2. Linking activity and identity, stressing the relevance of visualization

Because a major challenge in microbial ecology is to link identity and function, considerable efforts have been invested towards the development of techniques that allow this link. This topic has been the subject of thorough reviews in the last years [12,29–32], so we will only provide an overview of the techniques available and discuss how visualization is often determinant to understand the ecology of microbes. Most studies looking at single-cell activity in the ocean have focused on detecting protein or nucleic acid synthesis, traditionally measured using radioactive or stable-isotope labelled substrates. Initially, marine microbiologists used the capabilities of the flow cytometer to distinguish subpopulations of microbes (based on their size, nucleic acid content or pigment content) to flow sort these specific populations labelled with radio- or stable-isotopes [33] or specific dyes [34], sometimes including the downstream molecular characterization of the sorted populations (see [35] for a review on this topic).

MAR coupled with FISH has been the most widely used technique to link activity and function in the marine environment [11,22]. MAR-FISH (sometimes referred to as MAR-catalysed reporter deposition (CARD)-FISH when this variant of FISH is used, but also as STAR-FISH) has the advantages of substrate flexibility (any radio-labelled compound), high sensitivity, and that allows cells visualization, but the use of radioactive isotopes requires a specialized facility, and the processing of samples is destructive and relatively time consuming. Despite these disadvantages, the use of MAR-FISH has enabled, among many other things, the quantification of the contribution of certain bacterial and archaeal groups to different processes in the ocean [11] (and see [18,36,37] for an overview of MAR-FISH studies). An alternative non-radioactive method to visualize active cells is the use of the thymidine analogue bromodeoxyuridine (BrdU), followed by immunocytochemical detection of the DNA synthesizing cells [38], with further DNA analysis (16S-based diversity or metagenomics [39]) or FISH identification [15,40]. Yet, this technique is tedious, involves multiple steps that may result in sample loss, and there are some indications that BrdU might be toxic for some cells [41].

Another method used for linking identity and activity is stable-isotope-probing (SIP). It involves incubating an environmental sample with a stable-isotope labelled substrate, so that the active microorganisms can be identified by selective recovery and analyses of heavy-isotope enriched cellular components, like DNA, RNA, lipids or proteins (see [12,31,42] for reviews on this topic). It has been most commonly used for DNA (DNA-SIP) to elucidate which microbes are driving processes in the environment, like for instance methanol assimilation in coastal seawater [43]. SIP can also be combined with single-cell resolution techniques such as Raman micro-spectroscopy (Raman) and nanoscale secondary ion mass spectrometry (nanoSIMS). Raman is a vibrational spectroscopic method that allows chemical fingerprinting of individual cells [44]. It can be combined with FISH [45], and because the method is non-destructive, cells can undergo downstream analyses like targeted sorting of active cells for molecular characterization [46]. NanoSIMS is a destructive technique, but coupled with variations of FISH has provided very insightful information about metabolic fluxes within a symbioses [47], and the contribution of different microorganisms to fluxes of carbon and nitrogen in the environment [48–50]. Both Raman and nanoSIMS have high spatial resolution combined with sensitive quantification of the incorporated stable–isotope labelled compounds. The downside of both is that measurements are relatively low-throughput and dependent upon expensive instrumentation, most often available only at dedicated technical services. In addition, because of the oligotrophic nature of the ocean, incubations with stable-isotope labelled substrates are long, which may result in cross-feeding of labelled excreted products (i.e. labelled metabolites are released by the primary consumers and used by other microbes). This issue has been overcome in some studies with the use of heavy water (D_2_O), because deuterium can be used in lieu of hydrogen during lipid biosynthesis, and can be detected in prokaryotic cells within seconds after addition [46]. Moreover, the use of Raman for the sorting of deuterium-labelled live cells has recently been improved by increasing the throughput sorting potential from 1–2 sorted cells h^−1^ to 500 cells h^−1^ [51].

In the last years, click chemistry-based approaches like bioorthogonal non-canonical amino-acid tagging (BONCAT) have also arisen as a promising alternative to observe translationally active cells in the environment [16,17,52–54]. BONCAT uses synthetic amino acids (analogues for methionine) that upon incorporation can be fluorescently detected via copper-catalysed alkyne–azide click chemistry. It has the advantage that substrate concentrations in the micromolar range do not result in the induction of inactive cells, and the fluorescence intensity of the BONCAT signal correlates well with values of heterotrophic prokaryotic production [54]. BONCAT can be coupled with FISH [17,54], and with fluorescence-activated cell sorting (FACS) [53], so that follow-up molecular analyses to characterize the active populations can be performed.

Although the development of ‘omics approaches has provided an unprecedented way of looking at the microorganisms in the ocean and has unveiled metabolic processes that were hitherto unknown, inferring activity from genetic data is still extremely challenging. Visualization of the cells through microscopy or indirect techniques like flow cytometry add an extra layer of information that has often been shown to be essential to grasp the relevance of certain processes or the key players involved in these processes. Just a few examples of this postulate: most carbon fixation in the dark ocean was until recently attributed to Marine group I Thaumarchaeota given their numerical dominance in the dark ocean prokaryotic communities [55,56]. However, the combination of a variety of ’omics data with the observation and quantification of single-cell activity using MAR-FISH were determinant to conclude that low abundant nitrite-oxidizing bacteria have a major role in carbon fixation in the dark ocean, owing to the larger cell size and cell biomass of these bacteria compared to Thaumarchaeota [57]. Similarly, BONCAT-FISH and BONCAT-FACS were crucial to identify the key players in a microbial consortia catalysing the anaerobic oxidation of methane in deep methane seep sediments [53], which would have been impossible to tackle by non-targeted 'omics techniques, where the spatial resolution and the aggregation at the microscale is lost. Another nice example is the story behind the unusual endosymbiotic nitrogen-fixing cyanobacterium UCYN-A, that was initially discovered from short *nif*H sequences in the 1990s. It was not until the use of single-cell approaches like single-cell sorting and downstream molecular analyses together with the visualization through FISH (see [58] and references therein) that the ecology of this globally relevant symbiotic cyanobacterium was understood. Years later, nanoSIMS approaches were also decisive to unravel that UCYN-A plays a major role in the marine nitrogen cycle [49].

**Figure 2.**
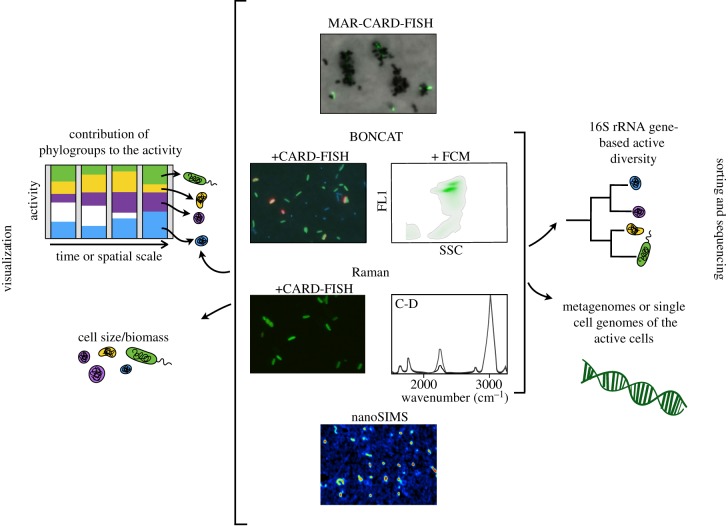
Diagram of cultured independent techniques that allow visualization of active microbes at the single-cell level. MAR relies in the incubation of a sample with a radio-labelled substrate, BONCAT with an artificial amino-acid (surrogate for methionine) and Raman and nanoSIMS with a stable-isotope labelled substrate (see text for details). These four techniques can be coupled with catalysed reporter deposition fluorescence *in situ* hybridization (CARD-FISH) to target-specific prokaryotic groups, which enables the quantification of the relative contribution of these groups to the activity. Visualization allows for the characterization of the cells in terms of cell size or biomass, and naturally occurring associations between cells. Among these single-cell techniques, only BONCAT and Raman can be coupled with the sorting of active populations for follow-up molecular characterization in terms of diversity (16S rRNA gene) or functional potential (single-cell amplified genomes or metagenomes of active cells). NanoSIMS image courtesy of Nestor Arandia and Anne Dekas.

So far only Raman and BONCAT approaches can be combined with techniques that allow direct visualization of the cells, with a broad phylogenetic characterization through CARD-FISH, and sorting of active subpopulations that can be used to assess their diversity (16S-rRNA gene sequencing) or their functional potential (metagenomes or single-cell amplified genomes, figure 2). Raman has the advantage over BONCAT that deuterium allows in principle tracking all active cells (autotrophs or heterotrophs), and it does not require cell fixation [51], whereas BONCAT relies on the ability of cells to take up methionine. On the other hand, BONCAT has a higher throughput that Raman, and thousands of cells can be analysed in a minute. Both these techniques open avenues in environmental research to tackle the role of prokaryotes in different biogeochemical cycles at the individual cell level, which is key for a better comprehension of microbial processes in the ocean.

## 3. Beyond the prokaryotic world

The importance of visualization to understand the ecology of microbes goes beyond the characterization of the active microbiome, and it is not only limited to the prokaryotic world. For instance, four different approaches have been used for quantifying nanoflagellate predation of prokaryotes and partitioning feeding rates into different groups of protists: (i) detection of stable-isotope labelled prey in the protist nucleic acids using SIP [59,60], (ii) flow cytometric separation of protistan groups based on size and fluorescence after feeding on radio-labelled bacterial tracers [61], (iii) microscopic observation of FISH-targeted protists feeding on fluorescently labelled bacterial tracers [62], and (iv) flow cytometric separation of single protist cells using a food vacuole stain and downstream phylogenetic fingerprinting of protists and preys [63]. The first of these approaches allowed molecular identification of the protists feeding on cyanobacteria and picoeukaryotes, but given the limitations of the method, the feeding rates could not be quantified. The second approach was used to quantify the rates by different cytometrically determined groups, but the method could not discriminate further. In the third of the approaches, the rates could be determined for different protistan groups via epifluorescence microscopy. With the fourth approach specific interactions between uncultured protists and their prey were untangled. Another example is provided in the work by Lima-Mendez *et al.* [64] that modelled the interaction between virus, prokaryotes and eukaryotes in the Global Ocean through co-occurrence networks using amplicon sequences and genomic data. In this case, microscopic observation was key to validate some of the network-generated hypotheses related to symbiosis, something that the molecular data alone could not do. Recently, a creative combination of a classical technique used in virology—the plaque assay—with advanced mass spectrometry imaging, has allowed visualization of the metabolic cross-talk between the coccolithophore *Emiliania huxleyi* and its virus at different stages of infection [65], unravelling a very heterogeneous and dynamic landscape of metabolic states. Similarly, transmission electronic microscopy has been crucial to unveiling that the production of membrane vesicles is a common trait in the three domains of life [66], broadening our view of how microbes interact in the ocean.

To summarize, visualization has been often decisive for understanding cell–cell interactions, and quantifying the role of marine microbes in global biogeochemical cycles. Although our field is experiencing a new era of discovery, with major breakthroughs derived from 'omics data, future works should try to integrate visualization if we want to gain a comprehensive view of how microbes contribute to the functioning of the marine ecosystem.

## Data accessibility

This article has no additional data.
